# DNase Promotes the Release of Sperm Trapped by NETs and Induces Important Changes in Donkey Sperm Motility Patterns

**DOI:** 10.3390/ani16142185

**Published:** 2026-07-14

**Authors:** Sandra Llorens, Sebastià Company, Luisina Chapero, Iván Yánez-Ortiz, Augusto Carluccio, Ippolito De Amicis, Jaime Catalán, Jordi Miró

**Affiliations:** 1 Equine Reproduction Service, Department of Animal Medicine and Surgery, Faculty of Veterinary Sciences, Autonomous University of Barcelona, ES-08193 Cerdanyola del Vallès, Spain; sandra.llorens@uab.cat (S.L.); sebastia.company@uab.cat (S.C.); 2 Department of Veterinary Medicine, University of Teramo, Località Piano d’Accio, IT-64100 Teramo, Italy; acarluccio@unite.it (A.C.); ideamicis@unite.it (I.D.A.); 3 Consejo Nacional de Investigaciones Científicas y Técnicas (CONICET), Centro Científico Tecnológico (CCT) Patagonia Confluencia, Neuquén 8300, Argentina; luisinachapero@gmail.com; 4 Faculty of Veterinary Sciences, National University of La Pampa, General Pico 6360, Argentina; 5 School of Veterinary Medicine, Faculty of Medical, Health and Life Sciences, International University of Ecuador, Quito 170411, Ecuador; ivan.yanez22@gmail.com; 6 Biotechnology of Animal and Human Reproduction (TechnoSperm), Institute of Food and Agricultural Technology, University of Girona, ES-17003 Girona, Spain; 7 Unit of Cell Biology, Department of Biology, Faculty of Sciences, University of Girona, ES-17003 Girona, Spain

**Keywords:** donkey, sperm, neutrophil extracellular traps (NETs), deoxyribonuclease (DNase), polymorphonuclear neutrophils (PMNs)

## Abstract

A deeper understanding of the donkey’s reproductive strategy is essential to improve reproductive efficiency in this species. In the jenny’s uterus, sperm selection relies on the post-insemination endometrial reaction, characterized by the influx of polymorphonuclear neutrophils (PMNs), which form extracellular traps (NETs) that capture spermatozoa. Of these, some are liberated through the action of a deoxyribonuclease (DNase) present in the seminal plasma. This study aimed to develop an in vitro model to evaluate the effects of NETs and DNase action on donkey sperm viability and motility. For this purpose, donkey ejaculates were collected, PMNs were isolated from the jenny’s blood and free spermatozoa were selected using a Silica gel filtration (EquiPure^®^, EP, Nidacon International AB, Mölndal, Sweden) to form five experimental groups: control (semen), SPZ-PMN, SPZ-PMN-DNase, SPZ-PMN-EP, SPZ-PMN-DNase-EP. Sperm viability and motility were assessed in all experimental groups using eosin-nigrosin staining and CASA sperm analysis system, respectively, at 1, 2 and 3 h of incubation. Sperm viability remained stable in the groups containing PMN, while it significantly (*p* < 0.05) decreased in the control group, suggesting a protective effect of PMN on sperm viability. Regarding kinetic parameters, spermatozoa initially captured by NETs and later liberated by DNase exhibited higher velocity (VCL, VSL, VAP), linearity (LIN), straightness (STR) and oscillation (WOB). These changes in sperm motility may be related to the physiological changes occurring before sperm capacitation. This study can improve the understanding of the unique reproductive strategy of donkeys and could be useful to improve in vitro sperm capacitation and embryo production.

## Simple Summary

After insemination, an important immune response occurs in the uterus of the female donkey. Some of the immune cells that migrate to the uterus form web-like structures (neutrophil extracellular traps; NETs) that temporarily capture sperm, which are later released by enzymes present in the seminal plasma. The aim of this study was to develop an in vitro model that reproduces NET formation and allows the evaluation of its effects on sperm survival and movement. The results showed that sperm incubated with these immune cells maintained viability for longer than sperm in semen alone. Moreover, sperm that were initially trapped and later released showed improved movement characteristics. These findings improve our understanding of the reproductive strategy of donkeys and may contribute to the development of more effective assisted reproduction techniques.

## 1. Introduction

For centuries, donkeys (*Equus asinus*) and mules, their hybrids with horses, have played a vital role in agriculture, transportation and warfare. However, the rise of mechanized farming and industrial development in the past century significantly decreased their populations and diminished their relevance. This shift placed many European donkey breeds at risk of disappearing and prompted the development of conservation and recovery initiatives. The effectiveness of these species conservation programs depends on well-structured recovery strategies that incorporate reproductive technologies adapted to the specific biological and physiological traits of each species [1].

The reproductive strategy varies among species, with variations in the site of semen deposition resulting in different barriers for sperm transport to the fertilization site [2]. In species of vaginal deposition, cervical mucus serves as the primary mechanism for sperm selection [3]. On the other hand, in species like horses or donkeys, where semen is directly deposited into the uterus, the sperm selection mechanism relies on the post-insemination endometrial reaction, characterized by a polymorphonuclear neutrophil (PMN) influx and the ability of these PMNs to form extracellular traps (NETs), which capture spermatozoa [4].

**Figure 1:**
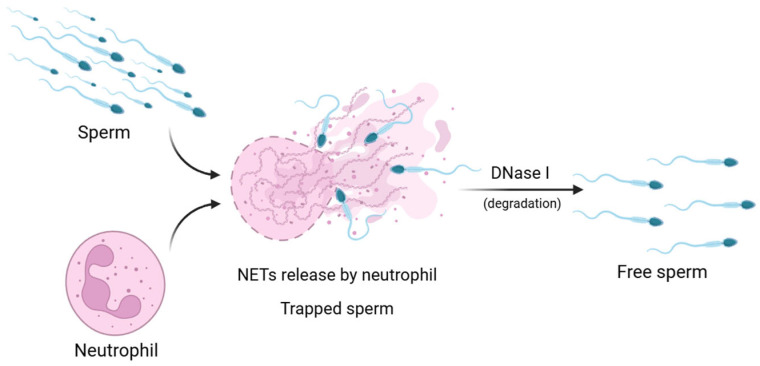
Formation of NETs (neutrophil extracellular traps), which are web-like structures of decondensed DNA, histones, and granular proteins. Spermatozoa trapped in these NETs could be released by the degradation of the net by the DNase I (image created with Biorender).

After semen deposition in donkeys, a physiological inflammation of the uterus occurs, in which many PMNs migrate to the uterine lumen and produce NETs [4]. The formation of these NETs involves the release of DNA from neutrophils building histone–DNA complexes along with granular components (such as elastase and myeloperoxidase) that, once outside the cell, create a NET where spermatozoa become trapped [5]. Both motile and immotile spermatozoa get trapped in the NETs, but the majority of them are viable and present high tail beating [6]. While seminal plasma is what induces the NETosis [7], it is also controlling the neutrophil influx and activity. Seminal plasma has been shown to reduce the physiological influx of neutrophils after semen deposition [8] and to allow some attached spermatozoa to become free [6]. Specifically, cysteine-rich secretory protein 3 (CRISP-3) isolated from seminal plasma and seminal DNase have been described to protect sperm from binding to neutrophils [9] and to release sperm from neutrophil extracellular traps [10] (Figure 1).

Deoxyribonuclease I (DNase I) is a Ca^2+^/Mg^2+^-dependent nucleic acid endonuclease which catalyzes the degradation of double-stranded DNA. It is the main nuclease found in serum, but it is also secreted into body fluids by various exocrine glands located along the gastrointestinal and urogenital tracts [11,12]. DNase has been found in the seminal plasma of several species [13,14,15,16,17], including stallions [2].

Although the formation of NETs and the activity of seminal DNase in degrading them have been described, the effect of this process on spermatozoa is still unknown and should be further investigated. Accordingly, the objective of this study was to develop an in vitro model in which spermatozoa are exposed to NETs and DNase activity. Afterward, free spermatozoa were selected by Silica Gel filtration (EquiPure^®^) to assess the sperm survival and motility characteristics. This study can improve the knowledge of equine reproductive strategies and could be useful for enhancing in vitro sperm capacitation and embryo production.

## 2. Materials and Methods

### 2.1. Animals

Three Catalan donkey (*Equus asinus*) jackasses, aged 6–12 years and with proven fertility, were used in this study. Previous studies of our group showed no differences in NET formation by PMNs between jennies [6,7]. Therefore, and as used previously [18], one jenny, aged 14 years, was used as a blood donor to obtain PMNs. All of them were housed at the Reproduction Service of the Department of Animal Medicine and Surgery at the Autonomous University of Barcelona located in Bellaterra, Spain. This facility is recognized by the European Union as an authorized center for equine sperm collection, operating under authorization number ES09RS01E. The animals were kept in paddocks, where males were housed individually and females in groups. They were fed a diet consisting of hay, grain and unlimited access to water. They were in good health, in accordance with the criteria established in Regulation (EU) 2016/429 of the European Parliament and of the Council dated 9 March 2016; consequently, both jackasses and jennies were free from equine infectious anemia, contagious equine metritis and equine viral arteritis. All experimental procedures were conducted in accordance with the ARRIVE guidelines and all applicable legislation regarding animal research, specifically Law 5/1995, Generalitat de Catalunya; Royal Decree 53/2013, Spain; and Directive 2010/63/EU of the European Parliament and of the Council. The study protocol was evaluated and approved by the Ethics Committee on Animal and Human Experimentation (CEEAH 1424) of the Universitat Autònoma de Barcelona, and no additional authorization number from the competent authority was required.

### 2.2. Isolation of PMNs

PMNs were isolated as described by Miró et al. [6]. Blood samples were collected from the jugular vein using BD Vacutainer tubes containing EDTA (18.0 mg ethylenediaminetetraacetic acid; BD, Plymouth, UK). These samples were incubated at 37 °C for 30 min to separate the foam, leukocyte-rich plasma and red blood cells. After incubation, the foam and plasma layers were removed using a suction pump, and the remaining supernatant from each sample was retained. This supernatant was then mixed in a 1:1 ratio with 0.02% EDTA solution (E8008; Sigma-Aldrich, St. Louis, MO, USA). A volume of 15 mL from this mixture was transferred to a 50 mL tube and combined with 15 mL of Ficoll Paque Plus (GE17-1440-02; Merck KGaA, Darmstadt, Germany). The resulting mixture was centrifuged at 500× *g* for 30 min at 20 °C (Medifriger BL-S; JP Selecta SA, Barcelona, Spain). Afterwards, the plasma layer, the monocyte-rich nebulous layer, and approximately half of the remaining transparent fraction above the red blood cell (RBC) pellet were carefully discarded, keeping the transparent layer containing the PMNs and the red blood cells. Next, 25 mL of phosphate-buffered saline (PBS, Sigma-Aldrich, St. Louis, MO, USA) was added to the tube containing the RBC and polymorphonuclear (PMN) cells. This suspension was then centrifuged at 500× *g* for 10 min at 20 °C. After centrifugation, the supernatant was removed. To lyse the remaining erythrocytes, 10 mL of lysis buffer (00-4333-57; ThermoFisher Scientific, Waltham, MA, USA) was added per milliliter of cell pellet. The mixture was gently agitated for 10 to 15 min to ensure complete lysis. Following this, 25 mL of PBS was added to stop the reaction, and the suspension was centrifuged once more at 500× *g* for 10 min at 20 °C. Finally, the supernatant was discarded, and the resulting cell pellet was resuspended in 1 mL of RPMI medium (R0883; Sigma-Aldrich, St. Louis, MO, USA) supplemented with 1% penicillin-streptomycin (P4333; Sigma-Aldrich, St. Louis, MO, USA). To determine the concentration of the isolated PMNs, the samples were analyzed using a flow cytometer (Sysmex XN-1000^TM^; Sysmex Corporation, Kobe, Japan), resulting in a mean ± SEM concentration of 46.9 ± 10.6 × 10^3^ cells/µL and a mean ± SEM neutrophil percentage of 71.6 ± 5.6.

### 2.3. Semen Collection and Process

A total of nine ejaculates (three per jackass) were collected using an artificial vagina (Hannover model, (Minitüb GmbH, Tiefenbach, Germany)), pre-warmed to a temperature between 48 and 50 °C and equipped with an in-line nylon filter to obtain gel-free semen samples. After collecting the ejaculate, 10 μL was taken to assess sperm concentration with a Neubauer chamber (Paul Marienfeld GmbH & Co. KG, Lauda-Königshofen, Germany). Raw semen was diluted 1:3 in a Kenney extender [19].

### 2.4. Evaluation of Sperm Viability

Sperm viability was evaluated using eosin-nigrosin staining [20]. For this purpose, a smear was prepared on a glass slide by mixing 10 μL of each semen sample with 10 μL of the staining solution, then dried at room temperature. A bright-field optical microscope (Carl Zeiss, Göttingen, Germany) was used at 1000× magnification with immersion oil to examine the samples. For each sample, at least 200 sperm cells were assessed, and the proportion of viable (eosin-negative) and non-viable (eosin-positive) spermatozoa was calculated.

### 2.5. Evaluation of Sperm Motility

**Figure 2:**
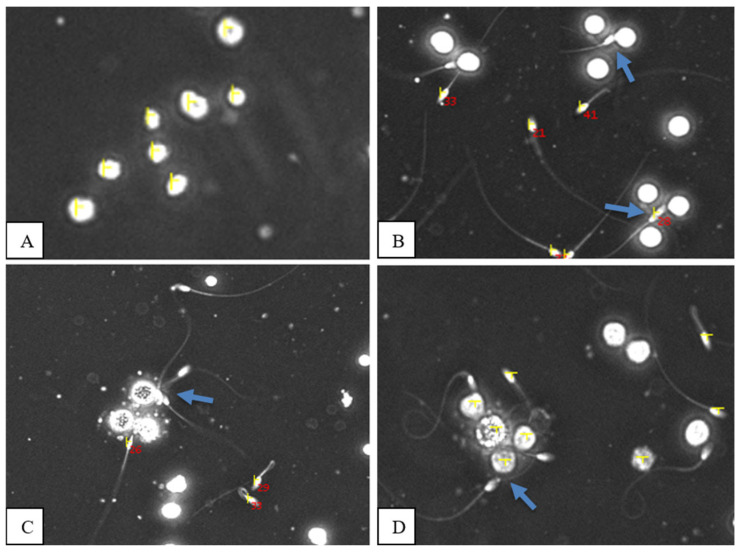
(**A**): CASA images of non-reactive PMN, the yellow lines indicate the cells detected by the CASA software. (**B**–**D**): PMN activation, with neutrophil extracellular trap (NET) formation, halo formation surrounding cells, and sperm aggregates (indicated by the blue arrow), progressively (**B**) < (**C**) < (**D**).

Sperm motility was evaluated using a computer-assisted sperm analysis (CASA) system (Integrated Semen Analysis System, ISAS^®^ Ver.1.0.15; Projects and Services R + D SL, Proiser; Valencia, Spain) (Figure 2). This system consisted of a negative phase-contrast microscope (Olympus BH-2, Tokyo, Japan) with a yellow light filter and a warm-up plate, and a digital video camera (Basler, Ahrensburg, Germany) connected to a computer containing the ISAS software. Samples were placed into a pre-warmed Spermtrack^®^ 10 chambers (Proiser R + D, Paterna, Spain) (37 °C) and examined at 200× magnification, with at least 500 spermatozoa in five different fields being counted per analysis. In each analysis, the following kinematic parameter were evaluated: curvilinear velocity (VCL, μm/s), straight line velocity (VSL, μm/s), average path velocity (VAP, μm/s), amplitude of lateral head displacement (ALH, μm), beat cross frequency (BCF, Hz), linearity coefficient (LIN, %), straightness coefficient (STR, %) and Wobble (WOB). The CASA system was configured according to the provider’s recommended settings: a frame rate of 25 images per second, connectivity set to 6, and particle area between 4 and 75 μm^2^. A minimum of 10 images was required to calculate ALH. Sperm were classified as motile if VAP was ≥10 μm/s, and as progressively motile if STR was ≥75%.

### 2.6. DNase

A DNase solution (10104159001, Sigma-Aldrich, St. Louis, MO, USA) was prepared using 1 mg of DNase per mL of Kenney’s extender (final concentration: 1 mg/mL), corresponding to 2000 U/mL. With respect to DNase cofactors, no supplemental Ca^2+^ or Mg^2+^ was included in the experimental medium.

### 2.7. Experimental Groups

For this study five treatments were prepared as follows:

- (SEMEN);
- (SPZ-PMN);
- (SPZ-PMN-DNase);
- (SPZ-PMN-EP);
- (SPZ-PMN-DNase-EP).

The first one consisted of fresh semen with a concentration between 300 and 350 × 10^6^ spz/mL (A), diluted 1:3 in Kenney’s extender, and the second one was a suspension of semen and PMNs. The sperm concentration was adjusted according to the concentration of isolated PMNs in each trial to obtain a sperm-to-PMN ratio of 3:1, resulting in the treatment (B) SPZ-PMN. The SPZ-PMN suspension was then divided to create the remaining experimental groups. The DNase solution was added to an extracted portion of the SPZ-PMN suspension at a 1:1 ratio to create the (C) SPZ-PMN-DNase treatment. All treatments were incubated in a water bath at 37 °C.

After 1 h of incubation, the SPZ-PMN-DNase and SPZ-PMN suspensions were analyzed. Aliquots from each sample were then processed using EquiPure (EPB-100, Nidacon International AB, Mölndal, Sweden) to separate spermatozoa from PMN cells, cellular debris, and NETs. Each aliquot was layered over the EquiPure, obtaining an EquiPure–semen ratio of 1:1.25. The samples were centrifuged at 300× *g* for 20 min at 20 °C to obtain the sperm filtrate, which was resuspended in 2 mL of Kenney’s extender, resulting in treatments (D) SPZ-PMN-EP and (E) SPZ-PMN-DNase-EP, respectively. The same procedure was applied to the samples after 2 and 3 h of incubation.

### 2.8. Statistical Analysis

All statistical analyses were performed using the R statistical software package (v.4.0.3, R Core Team; Vienna, Austria), and graphs were generated with GraphPad Prism (v.8.4.0, GraphPad Software LLC; San Diego, CA, USA). Shapiro–Wilk tests were applied to verify the normal distribution of the data, and Levene’s tests were used to assess homoscedasticity of variances. When necessary, data were transformed using the arcsine √x transformation to meet parametric assumptions. Results are expressed as means ± standard error of the mean (SEM). Motile sperm subpopulations were identified through non-hierarchical multivariate cluster analysis using the k-means model based on Euclidean distances calculated from the kinematic parameters (VCL, VSL, VAP, LIN, STR, WOB, ALH, and BCF) of each spermatozoon. The effect of treatments (Control, SPZ-PMN, SPZ-PMN-DNase, SPZ-PMN-DNase-EP, and SPZ-PMN-EP) was compared using a generalized linear mixed model with repeated measures over time, where the fixed effect factor was the treatment, the within-subject factor was the incubation time (1, 2, and 3 h), and the random effect factor was the donkey. Ejaculates obtained from the jackasses were considered the biological replicates in this study. In all analyses, pairwise comparisons were performed using Bonferroni post hoc tests, with a minimum level of statistical significance set at *p* ≤ 0.05.

## 3. Results

### 3.1. Sperm Viability

**Figure 3:**
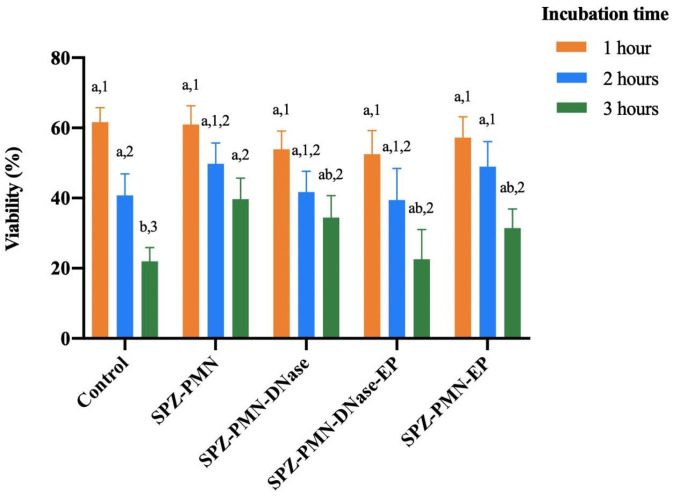
Sperm viability (%) in the 5 experimental groups (SEMEN, SPZ-PMN, SPZ-PMN-DNase, SPZ-PMN-DNase-EP, SPZ-PMN-EP) throughout the incubation time (1, 2, 3 h). Different superscripts (a, b) indicate significant (*p* < 0.05) differences between experimental groups within a given time. Different numbers (1, 2, 3) mean significant (*p* < 0.05) differences between times within a given experimental group. Data are presented as mean ± SEM of nine independent replicates.

The analysis of the results of sperm viability (Figure 3) showed significant differences (*p* < 0.05) between the control treatment and the PMN–SPZ treatment after 3 h of incubation.

In addition, in the control group (spermatozoa alone, SPZ), viability decreased significantly (*p* < 0.05) between 1 and 2 h, as well as between 2 and 3 h of incubation. This pattern was not observed in the PMN treatments, in which a significant decrease was only detected between 1 and 3 h of incubation.

### 3.2. Sperm Motility

#### 3.2.1. Total and Progressive Motility

**Figure 4:**
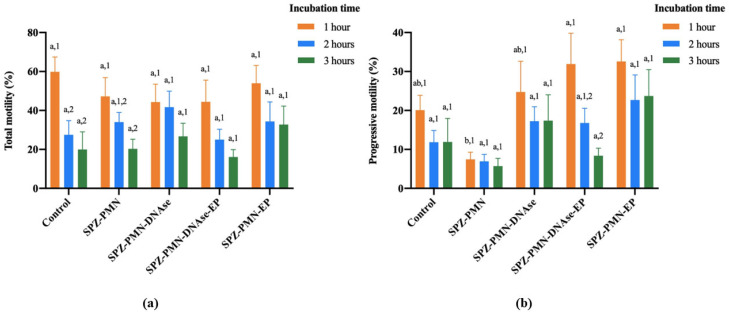
Total motility (%) (**a**) and progressive motility (%) (**b**) in the 5 experimental groups (SEMEN, SPZ-PMN, SPZ-PMN-DNase, SPZ-PMN-DNase-EP, SPZ-PMN-EP) throughout the incubation time (1, 2, 3 h). Different superscripts (a, b) indicate significant (*p* < 0.05) differences between experimental groups within a given time. Different numbers (1, 2) mean significant (*p* < 0.05) differences between times within a given experimental group. Data are presented as mean ± SEM of nine independent replicates.

As shown in Figure 4a, no differences in total sperm motility were observed among the experimental groups at the different incubation times (1, 2, and 3 h). The control group exhibited a significant decrease (*p* < 0.05) at 2 and 3 h compared with 1 h of incubation. However, none of the experimental groups showed differences between 1 and 2 h, and the SPZ–PMN group only exhibited significant differences between 1 and 3 h of incubation.

Regarding the progressive motility, shown in Figure 4b, the SPZ-PMN-DNase, SPZ-PMN-DNase-EP and SPZ-PMN-EP groups showed the highest percentages of motile sperm at 1 h, with no statistically significant differences compared to 2 h of incubation. However, the SPZ-PMN-DNase-EP group evidenced a significant decrease at 3 h of incubation. The values of progressive motility remained stable throughout the incubation time in the SPZ-PMN-DNase group. The lowest motile sperm percentages are in the SPZ-PMN group, at all incubation times, and significantly lower than other treatments at 1 h (*p* < 0.05). The control group showed a decline in the progressive motility over time, but with no statistically significant differences.

#### 3.2.2. Velocity Parameters

**Figure 5:**
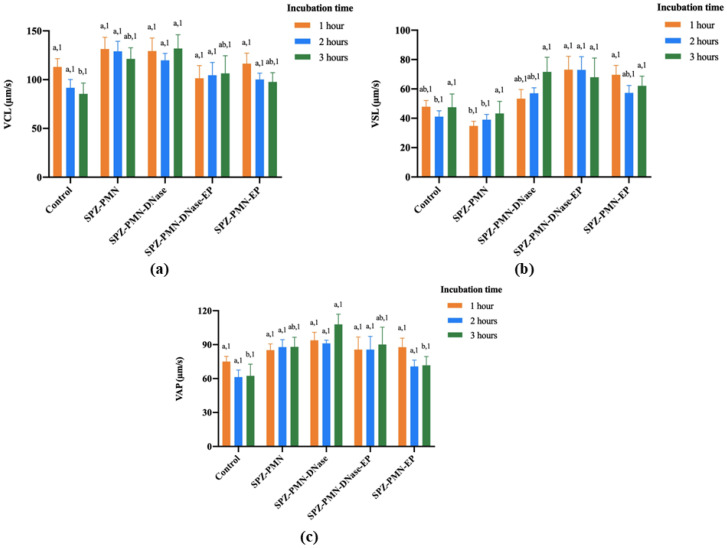
Curvilinear velocity (VCL, μm/s) (**a**), straight line velocity (VSL, μm/s) (**b**) and average path velocity (VAP, μm/s) (**c**) in the 5 experimental groups (SEMEN, SPZ-PMN, SPZ-PMN-DNase, SPZ-PMN-DNase-EP, SPZ-PMN-EP) throughout the incubation time (1, 2, 3 h). Different superscripts (a, b) indicate significant (*p* < 0.05) differences between experimental groups within a given time. Number (1) in different figures mean significant (*p* < 0.05) differences between times within a given experimental group. Data are presented as mean ± SEM of nine independent replicates.

The velocity parameters, including VCL, VSL and VAP are shown in Figure 5, respectively.

Regarding VCL (Figure 5a), statistically significant differences were observed (*p* < 0.05), with lower VCL values after 3 h of incubation in the control group compared with the SPZ–PMN–DNase treatment. No significant differences were detected among the different incubation times within each treatment.

With respect to VSL (Figure 5b), the groups treated with DNase and/or EP exhibited the highest values. Significant differences were observed, with lower VSL at 1 h of incubation in the SPZ–PMN treatment compared with the SPZ–PMN–DNase–EP and SPZ–PMN–EP treatments. In addition, after 2 h of incubation, the control and SPZ–PMN treatments showed significantly lower VSL values (*p* < 0.05) compared with the SPZ–PMN–DNase–EP treatment. No significant differences (*p* > 0.05) were observed among incubation times within each treatment.

Analysis of the VAP results (Figure 5c) showed that the SPZ–PMN–DNase and SPZ–PMN–DNase–EP groups exhibited consistently high values at all incubation times. Significant differences were observed after 3 h of incubation, with lower VAP values in the control and SPZ–PMN–EP treatments compared with the SPZ–PMN–DNase treatment. No significant differences (*p* > 0.05) were detected among incubation times within each treatment.

#### 3.2.3. Linearity

The SPZ-PMN-DNase-EP and SPZ-PMN-EP groups showed the highest (*p* < 0.05) LIN values at all evaluated time points, with no statistically significant changes over time within each treatment (*p* > 0.05). However, significant differences among treatments were detected at the different incubation times (*p* < 0.05).

Specifically, after 1 h of incubation, lower LIN values were observed in the control and SPZ–PMN treatments compared with the SPZ–PMN–DNase–EP treatment, as well as lower LIN values in the SPZ–PMN treatment compared with the SPZ–PMN–DNase–EP and SPZ–PMN–EP treatments.

Similar results were observed after 2 h of incubation, with significantly lower LIN values (*p* < 0.05) in the control and SPZ–PMN treatments compared with the SPZ–PMN–DNase–EP treatment, and lower LIN values in the SPZ–PMN treatment compared with the SPZ–PMN–DNase–EP and SPZ–PMN–EP treatments.

**Figure 6:**
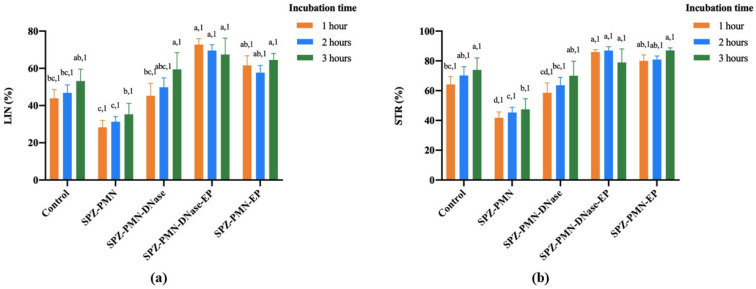
Linearity (LIN, %) (**a**) and straightness (STR, %) (**b**) in the 5 experimental groups (SEMEN, SPZ-PMN, SPZ-PMN-DNase, SPZ-PMN-DNase-EP, SPZ-PMN-EP) throughout the incubation time (1, 2, 3 h). Different superscripts (a, b, c, d) indicate significant (*p* < 0.05) differences between experimental groups within a given time. Number (1) in different figures mean significant (*p* < 0.05) differences between times within a given experimental group. Data are presented as mean ± SEM of nine independent replicates.

Finally, after 3 h of incubation, significant differences were detected, with lower LIN values in the SPZ–PMN treatment compared with the SPZ–PMN–DNase, SPZ–PMN–DNase–EP, and SPZ–PMN–EP treatments (Figure 6a).

#### 3.2.4. Straightness

Regarding straightness (Figure 6b), the results showed that the SPZ–PMN–DNase–EP and SPZ–PMN–EP treatments exhibited the highest values at all incubation times, with no significant differences over time within each treatment. However, as observed for LIN, significant differences among groups were detected at the evaluated time points.

Specifically, after 1 h of incubation, significantly lower STR values (*p* < 0.05) were observed in the SPZ–PMN treatment compared with the control, SPZ–PMN–DNase–EP, and SPZ–PMN–EP treatments. In addition, significantly lower STR values were detected in the control and SPZ–PMN–DNase treatments compared with the SPZ–PMN–DNase–EP treatment, as well as lower STR values in the SPZ–PMN–DNase treatment compared with the SPZ–PMN–EP treatment.

After 2 h of incubation, significant differences were observed, with lower STR values in the SPZ–PMN treatment compared with the control, SPZ–PMN–DNase–EP, and SPZ–PMN–EP treatments. Moreover, the SPZ–PMN–DNase treatment showed significantly lower STR values than the SPZ–PMN–DNase–EP treatment.

Finally, after 3 h of incubation, significant differences were detected, with lower STR values in the SPZ–PMN treatment compared with the control, SPZ–PMN–DNase–EP, and SPZ–PMN–EP treatments.

#### 3.2.5. Wobble

**Figure 7:**
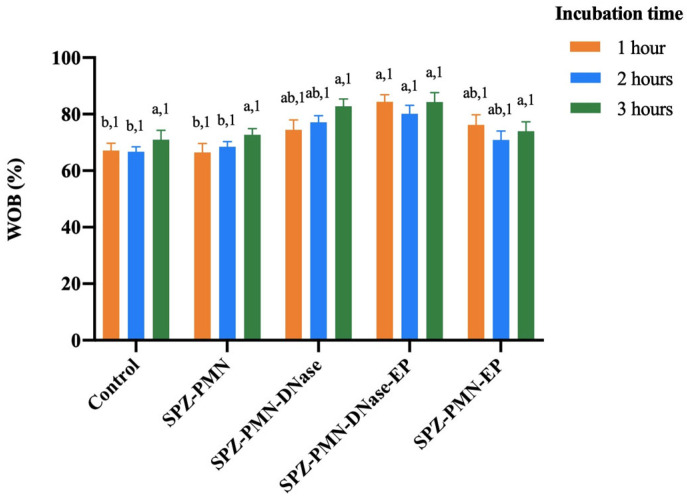
Wobble (WOB, %) in the 5 experimental groups (SEMEN, SPZ-PMN, SPZ-PMN-DNase, SPZ-PMN-DNase-EP, SPZ-PMN-EP) throughout the incubation time (1, 2, 3 h). Different superscripts (a, b) indicate significant (*p* < 0.05) differences between experimental groups within a given time. Number (1) in different figures mean significant (*p* < 0.05) differences between times within a given experimental group. Data are presented as mean ± SEM of nine independent replicates.

Wobble is presented in Figure 7. The SPZ-PMN-DNase-EP group showed the highest Wobble values across all incubation times, with no statistically significant variations over time within the same treatment.

Significant differences were observed after 1 h of incubation, with lower WOB values in the control and SPZ–PMN treatments compared with the SPZ–PMN–DNase–EP treatment. Similar results were observed after 2 h of incubation, with significantly lower WOB values (*p* < 0.05) in the control and SPZ–PMN treatments compared with the SPZ–PMN–DNase–EP treatment.

#### 3.2.6. Lateral Head Displacement

**Figure 8:**
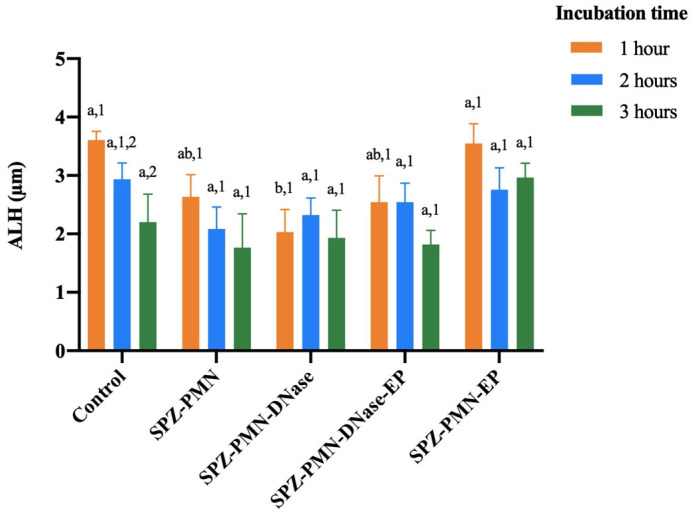
Lateral head displacement (ALH, μm) in the 5 experimental groups (SEMEN, SPZ-PMN, SPZ-PMN-DNase, SPZ-PMN-DNase-EP, SPZ-PMN-EP) throughout the incubation time (1, 2, 3 h). Different superscripts (a, b) indicate significant (*p* < 0.05) differences between experimental groups within a given time. Different numbers (1, 2) mean significant (*p* < 0.05) differences between times within a given experimental group. Data are presented as mean ± SEM of nine independent replicates.

The results for lateral head displacement (Figure 8) showed a significant decrease (*p* < 0.05) within the Control treatment between 1 and 3 h of incubation.

In addition, a significant decrease (*p* < 0.05) in lateral head displacement was observed in the SPZ–PMN–DNase treatment compared with the Control and SPZ–PMN–EP treatments after 1 h of incubation.

#### 3.2.7. Beat Cross Frequency

**Figure 9:**
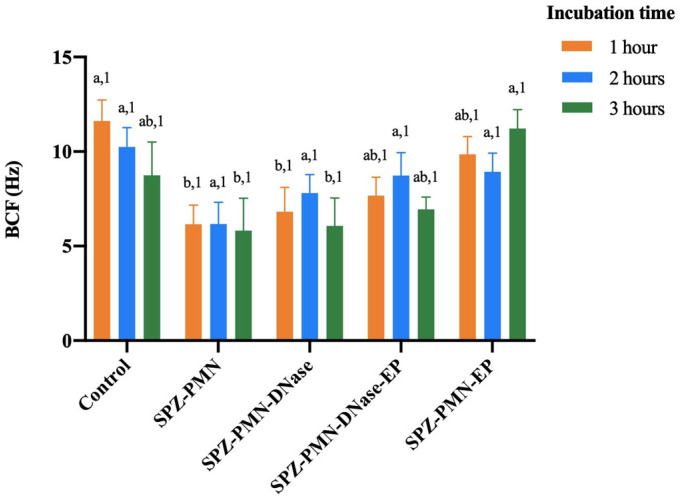
Beat cross frequency (BCF, Hz) in the 5 experimental groups (SEMEN, SPZ-PMN, SPZ-PMN-DNase, SPZ-PMN-DNase-EP, SPZ-PMN-EP) throughout the incubation time (1, 2, 3 h). Different superscripts (a, b) indicate significant (*p* < 0.05) differences between experimental groups within a given time. Number (1) in different figures mean significant (*p* < 0.05) differences between times within a given experimental group. Data are presented as mean ± SEM of nine independent replicates.

Regarding the Beat Cross Frequency (Figure 9) no significant differences were observed within the same treatment among the different incubation times. However, significant differences among treatments were detected.

Specifically, after 1 h of incubation, significantly lower BCF values (*p* < 0.05) were observed in the SPZ–PMN and SPZ–PMN–DNase treatments compared with the Control treatment, but not when compared with the EP-treated groups. In addition, after 3 h of incubation, significantly lower BCF values (*p* < 0.05) were detected in the SPZ–PMN and SPZ–PMN–DNase treatments compared with the SPZ–PMN–EP treatment.

### 3.3. Motile Sperm Subpopulation Study

As shown in Table 1, four different motile sperm subpopulations (SP1, SP2, SP3, SP4) were identified in all treatments. SP1 exhibited the lowest values for all velocity parameters (VCL, VSL and VAP). In contrast, SP3 showed the highest mean velocities, together with the greatest straightness, linearity and oscillation (WOB). SP4 displayed the highest VCL and ALH but reduced linearity and straightness. BCF remained relatively consistent among subpopulations.

**Figure 10:**
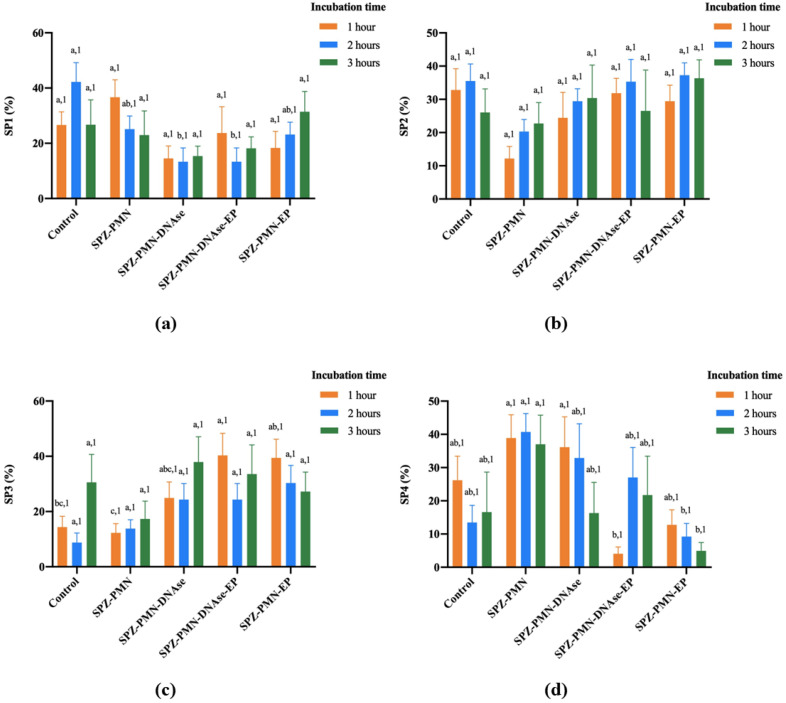
Percentages of motile sperm subpopulations (SP1 (**a**), SP2 (**b**), SP3 (**c**), SP4 (**d**)) in the 5 experimental groups (SEMEN, SPZ-PMN, SPZ-PMN-DNase, SPZ-PMN-DNase-EP, SPZ-PMN-EP) throughout the incubation time (1, 2, 3 h). Different superscripts (a, b, c) indicate significant (*p* < 0.05) differences between experimental groups within a given time. Number (1) in different figures mean significant (*p* < 0.05) differences between times within a given experimental group. Data are presented as mean ± SEM of nine independent replicates.

The proportion of sperm belonging to each motile subpopulation (SP1–SP4) was affected by the different experimental conditions and incubation times (Figure 10). Treatments containing DNase significantly (*p* < 0.05) decreased the proportion of sperm belonging to SP1 (Figure 10a), the slowest subpopulation, at 2 h of incubation. The proportion of SP2 remained stable among treatments and incubation times, with no statistically significant differences (Figure 10b). In contrast, SP3, the subpopulation with highest velocity, straightness, linearity and wobble, showed marked treatment-related variations. The control and the SPZ-PMN group had the lowest proportion at 1 h of incubation, DNase treatments, with or without EP, tend to have a higher percentage of spermatozoa included in SP3 (Figure 10c). In the case of SP4 (Figure 10d), the EP-treated groups showed a lower percentage of this subpopulation (*p* < 0.05). Specifically, after 1 h of incubation, the SPZ–PMN–DNase–EP treatment exhibited a lower percentage of SP4 compared with the SPZ–PMN and SPZ–PMN–DNase treatments. On the other hand, after 2 and 3 h of incubation, the SPZ–PMN–EP treatment showed a lower percentage of SP4 compared with the SPZ–PMN treatment.

## 4. Discussion

Considering that sperm selection in the equine uterus is mediated by PMN infiltration and NET formation [4], with seminal plasma DNase potentially contributing to the release of trapped spermatozoa [10], this study provides, to the best of our knowledge, the first attempt to characterize the effects of NETs and DNase on the kinematic parameters and viability of donkey sperm.

Sperm viability data showed a decline over the incubation period in all treatments. However, whereas viability in the control group (SPZ) decreased significantly between 1 and 2 h and again between 2 and 3 h of incubation, in the treatments containing PMNs, with or without DNase, a significant reduction was only detected between 1 and 3 h. Furthermore, after 3 h of incubation, viability was significantly lower in the control group than in the PMN–SPZ treatment. This finding suggests that the union with PMNs has a protective effect on spermatozoa’s viability, which is consistent with the observations reported by Miró et al. [6].

Regarding sperm motility, our results show that the control group, spermatozoa alone, had a significant (*p* < 0.05) decrease in total motility at 2 h of incubation but not the experimental groups with PMNs. On the other hand, the SPZ-PMN group exhibited the lowest percentages of progressive motile sperm across all incubation times, statistically significant (*p* < 0.05) at 1 h of incubation in relation to other experimental groups. These findings are in agreement with those reported by Wei et al. [21], who observed a decrease in sperm motility upon interaction with PMN in goats. As spermatozoa entrapped by NETs are immobilized [10], thus preventing forward progression, a lower proportion of progressive motility was expected.

With respect to velocity descriptors, the treatments including DNase (SPZ–PMN–DNase, SPZ–PMN–DNase–EP) maintained higher velocity parameters (VCL, VSL, VAP). This is evident in the VCL for the SPZ–PMN–DNase treatment compared with the control, in the VSL for the SPZ–PMN–DNase–EP treatment compared with the control and SPZ, and in the VAP for the SPZ–PMN–DNase treatment compared with the control. Overall, treatments involving DNA or EP appeared to help maintain higher sperm velocity parameters over time. Moreover, the addition of DNase alone maintained or decreased linearity and straightness compared to the control group. In contrast, groups filtered with EP had the highest linearity and straightness values, in accordance with the observations of Ortiz et al. [22,23] and Papas et al. [24]. To be able to pass through the colloid, spermatozoa need to line up in the direction of the centrifugal force and move through the colloid–semen interface, which may explain the increased linearity observed in these groups filtered with EP. The combination of DNase and EP also resulted in consistently higher oscillation (WOB) values throughout incubation. In contrast, the control and SPZ-PMN groups exhibited lower WOB values. Concerning ALH, it decreased significantly (*p* < 0.05) over time in the control group, whereas it remained stable in treatments containing PMN, DNase or EP. In relation to BCF, the PMN and DNase treatments without EP exhibited the lowest values at 1 and 3 h, followed by a gradual, non-significant decline over time. Overall, spermatozoa initially entrapped by PMNs and later released by the action of the DNase showed improved kinematic performance, characterized by higher velocity, linearity, straightness, and wobble.

The study of motile sperm subpopulations further confirms these findings. Four different motile sperm subpopulations (SP1–SP4) were identified in the present study. Distinct motile sperm subpopulations exhibiting specific kinematic patterns have been previously identified in donkeys [25,26,27]. These groups of spermatozoa have also been reported in the ejaculates of other mammalian species from diverse phylogenetic origins, such as gazelles [28], dogs [29], horses [30], red deer [31], donkey [32], cattle [33], boars [34] and sheep [35]. In the present work, the distribution of these subpopulations varied significantly (*p* < 0.05) depending on the treatment and incubation time. The experimental groups containing DNase significantly (*p* < 0.05) reduced the proportion of the slow-moving sperm subpopulation (SP1) and increased the proportion of the highly motile and linear subpopulation (SP3), particularly after 1–2 h of incubation.

Based on these results, we can hypothesize that the main part of motile spermatozoa is initially captured by NETs, which would explain the lower motility observed in the SPZ–PMN group. In this group, the motility assessment corresponds to the remaining free spermatozoa, which display poorer kinematic parameters. In the presence of DNase, however, the degradation of NETs releases the previously trapped spermatozoa, increasing the proportion of spermatozoa with better kinetic parameters. These findings suggest that sperm–neutrophil interaction and DNase activity may function as a selective mechanism in donkey sperm, in agreement with the hypothesis proposed by Doty et al. [36]. The observed changes in sperm motility patterns may be associated with the modifications that take place prior to sperm capacitation.

Both capacitation and hyperactivation form part of a continuous physiological progression enabling sperm to fertilize the oocyte [37]. The hyperactivation stallion sperm movement is characterized by a decrease in LIN and STR and increase in ALH [38]. This pattern of movement has also been described in other species [39,40,41,42]. This hyperactivated state is typified by vigorous, high-amplitude, and asymmetric flagellar beating and occurs as spermatozoa approach the oocyte [43]. But, throughout most of their passage within the female reproductive tract, spermatozoa display a flagellar motion of low amplitude and high frequency that supports progressive, linear movement [44]. This motility pattern is consistent with that described in the results of the present study, once spermatozoa are released from the PMNs by the action of DNase, and may be associated with pre-capacitation changes.

Silica gel (EP) filtration can be a method to isolate incubated spermatozoa with PMNs; however, considering that EP filtration can induce some motility pattern changes with an increase in LIN and STR, further research is needed to confirm if this method is the most suitable.

This study contributes to a better understanding of the specific reproductive strategies of donkeys and provides valuable information for optimizing in vitro sperm capacitation and embryo production. However, further investigation is needed assess the impact of PMNs and DNase on sperm capacitation, which may have important implications for improving the efficiency of assisted reproduction techniques in this species.

## 5. Conclusions

In conclusion, the interaction between spermatozoa and polymorphonuclear neutrophils (PMNs) appears to play a relevant role in maintaining sperm viability over time, suggesting that PMNs could act as a reservoir for spermatozoa. Once sperm are released, either through DNase activity or simple filtration with EquiPure, they have higher progressive motility. Moreover, sperm entrapped by PMNs and subsequently released by DNase show improved kinematic parameters, including higher velocity, linearity straightness, and wobble. These findings support the hypothesis that NETs and DNase may function as a selective mechanism in donkey sperm, inducing changes that could lead to sperm capacitation.

## Figures and Tables

**Table 1:** Descriptive parameters (mean ± SEM and range) of the four sperm subpopulations (SP1, SP2, SP3, SP4) identified in donkey semen.

	SP1	SP2	SP3	SP4
*n*	2399	3062	1985	2480
	Mean ± SEM	Mean ± SEM	Mean ± SEM	Mean ± SEM
**VCL**	39.92 ± 0.45	106.19 ± 0.36	148.76 ± 0.55	164.26 ± 0.71
**VSL**	14.76 ± 0.21	63.31 ± 0.28	98.99 ± 0.41	33.31 ± 0.35
**VAP**	22.27 ± 0.27	79.92 ± 0.27	119.72 ± 0.38	102.55 ± 0.41
**LIN**	38.18 ± 0.42	61.20 ± 0.30	68.16 ± 0.35	20.63 ± 0.21
**STR**	64.27 ± 0.48	79.51 ± 0.25	82.89 ± 0.25	33.03 ± 0.34
**WOB**	55.84 ± 0.37	76.37 ± 0.23	81.57 ± 0.27	63.52 ± 0.22
**ALH**	1.78 ± 0.02	3.50 ± 0.02	4.43 ± 0.04	6.09 ± 0.03
**BCF**	7.17 ± 0.06	10.04 ± 0.06	10.09 ± 0.08	9.89 ± 0.08

SEM: standard error of the mean; VCL (μm/s): curvilinear velocity; VSL (μm/s): straight line velocity; VAP (μm/s): average path velocity; LIN (%): linearity; STR (%): straightness; WOB (%): wobble; ALH (μm): amplitude of lateral head displacement; BCF (Hz): beat-cross frequency.

## Data Availability

The datasets supporting the conclusions of this article are available from the corresponding authors on reasonable request.

## Abbreviations

The following abbreviations are used in this manuscript:

PMN/PMNs	Polymorphonuclear neutrophils
NET/NETs	Neutrophil extracellular traps
DNase	Deoxyribonuclease
DNA	Deoxyribonucleic acid
SPZ	Spermatozoa
EP	EquiPure
RBC	Red blood cells
PBS	Phosphate-buffered saline
RPMI	Roswell Park Memorial Institute medium
EDTA	Ethylenediaminetetraacetic acid
CASA	Computer-Assisted Sperm Analysis
ISAS	Integrated Semen Analysis System
VCL	Curvilinear Velocity
VSL	Straight Line Velocity
VAP	Average Path Velocity
ALH	Amplitude of Lateral Head displacement
BCF	Beat Cross Frequency
LIN	Linearity
STR	Straightness
WOB	Wobble
SEM	Standard Error of the Mean
SP1/SP2/SP3/SP4	Sperm subpopulations 1–4
CRISP-3	Cysteine-Rich Secretory Protein 3
